# Remote monitoring of chronic heart failure patients: invasive versus non-invasive tools for optimising patient management

**DOI:** 10.1007/s12471-019-01342-8

**Published:** 2019-11-19

**Authors:** J. F. Veenis, J. J. Brugts

**Affiliations:** Thorax Centre, Erasmus MC, University Medical Centre Rotterdam, Rotterdam, The Netherlands

**Keywords:** Remote monitoring, Heart failure, CardioMEMS, Telemedicine, Telemonitoring, eHealth

## Abstract

Exacerbations of chronic heart failure (HF) with the necessity for hospitalisation impact hospital resources significantly. Despite all of the achievements in medical management and non-pharmacological therapy that improve the outcome in HF, new strategies are needed to prevent HF-related hospitalisations by keeping stable HF patients out of the hospital and focusing resources on unstable HF patients. Remote monitoring of these patients could provide the physicians with an additional tool to intervene adequately and promptly. Results of telemonitoring to date are inconsistent, especially those of telemonitoring with traditional non-haemodynamic parameters. Recently, the CardioMEMS device (Abbott Inc., Atlanta, GA, USA), an implantable haemodynamic remote monitoring sensor, has shown promising results in preventing HF-related hospitalisations in chronic HF patients hospitalised in the previous year and in New York Heart Association functional class III in the United States. This review provides an overview of the available evidence on remote monitoring in chronic HF patients and future perspectives for the efficacy and cost-effectiveness of these strategies.

## Introduction

The management of patients with chronic heart failure (HF) places a high burden on health care resources due to the frequent follow-up visits combined with recurrent hospitalisations due to cardiac decompensation [1]. Early detection of HF deterioration is crucial to prevent HF-related hospitalisations, potentially improve overall survival and quality of life and lower the burden on health care resources. Remote monitoring of chronic HF patients can aid in the detection of HF deterioration; therefore several remote monitoring strategies have been developed. In this review, we provide an overview of available evidence on remote monitoring of chronic HF patients and provide further perspectives of anticipated developments in the remote care of HF.

### Non-haemodynamic remote monitoring

Over the last few decades, several studies have investigated the use of non-haemodynamic remote monitoring. However, the results have been largely inconsistent. A recently updated Cochrane review included 41 randomised controlled trials (RCTs) investigating the use of structured telephone support (25 studies, 9332 patients) or non-invasive telemonitoring (18 studies, 3860 patients) compared with standard HF care [2]. This review showed a modest beneficial effect of remote monitoring on all-cause mortality and HF-related hospitalisations, although no effect on the overall hospitalisation rates was observed. However, the quality of the evidence of this review is limited by the many different inclusion and exclusion criteria for patients included in the studies and considerably heterogeneity of compared data. Also, the studies included used different intervention therapies, ranging from telephone calls only, weight monitoring to complex multiple-variable telemonitoring strategies making it difficult to conclude which component drives the effect. Additionally, the majority of selected individual studies (more than twenty) were neutral.

Multiple large multi-centre prospective clinical studies and RCTs have investigated multiple non-invasive remote monitoring strategies, ranging from symptom and body weight monitoring to complex and intensive strategies including body weight, blood pressure, electrocardiography and peripheral capillary oxygen saturation. The landmark studies of high quality design and well specified intervention show no consistent beneficial effect of non-haemodynamic remote monitoring in HF patients (Tab. 1; [3–11]). Of specific note and most promising are the recent results of TIM-HF2 trial showing a benefit on all-cause mortality and cardiovascular hospitalizations of a well structured but labour intensive 24/7 telemonitoring strategy, but remarkably showed no effect on quality of life [6]. Also, ‘real-world’ data, such as those from the Medicare database, did not show consistent benefits of non-haemodynamic remote monitoring strategies on mortality or hospitalisation rates [12]. Our conclusion is that although results are inconsistent for non-invasive telemonitoring, the simplicity makes it potentially useful for larger groups of HF patients at relatively lower risk or less symptomatic, where invasive telemonitoring may have more impact in sicker patients.

**Table 1:** Non-invasive remote monitoring in heart failure (*HF*) patients^a^

Trial/study	Author; journal; year	No. of patients	Parameter	Endpoint	Impact on HF hospitalisation
TEN-HMS [5]	Cleland et al.; J Am Coll Cardiol; 2005	426	Signs/symptoms, daily weights, BP, nurse telephone calls	HF hospitalisation	Non-significant
TELE-HF [4]	Chaudhry et al.; N Engl J Med; 2010	1653	Signs/symptoms, daily weights	HF hospitalisation	Non-significant
TIM-HF [7]	Koehler F et al.; Circulation; 2011	710	Signs/symptoms, daily weights	HF hospitalisation	Non-significant
INH [3]	Angermann et al.; Circ Heart Fail; 2012	715	Signs, symptoms, telemonitoring nurse coordinated	HF hospitalisation	Non-significant
WISH [10]	Lynga et al.; Eur J Heart Fail; 2012	344	Daily weights	HF hospitalisation	Non-significant
CHAT [9]	Krum et al.; Cardiovasc Ther; 2013	405	Monthly telephone‐based automated telemedicine system	HF hospitalisation	Non-significant
BEAT-HF [11]	Ong et al.; JAMA Intern Med; 2016	1437	Signs, symptoms, daily weights, nurse communications	HF hospitalisation	Non-significant
TIM-HF2 [6]	Koehler F et al.; Lancet; 2018	1571	Web-based remote monitoring on daily weight, BP, pulse, ECG, peripheral capillary oxygen saturation, a self-related health status. ECG and BP machine at home	Reduction in the weighted average of ‘the % of days lost due to unplanned CV hospital admissions or death’	HR 0.80; 95% CI 0.65–1.00

*BP* blood pressure, *CV* cardiovascular, *ECG* electrocardiography

^a^Demonstrating the landmark trials only, sample size >250 patients, discounting studies with only phone calls as intervention

### Remote monitoring using pacemaker/ICD devices

Multiple studies have investigated the remote monitoring abilities of implantable cardioverter defibrillator/cardiac resynchronisation therapy (ICD/CRT) devices in chronic HF patients to improve HF-related hospitalisation rates (Tab. 2). The MORE-CARE multi-centre RCT showed that remote monitoring of advanced diagnostics via CRT‑D did not reduce mortality or hospitalisation rates, although the health care resource utilisation was reduced due to a reduction in outpatient follow-up visits [13]. Additionally, the DOT-HF, OptiLink and REM-HF trials investigated the use of remote monitoring using ICD/CRT devices, but all failed to show a reduction in HF-related hospitalisation rates [14–16]. The DOT-HF trial even showed an increase in the number of HF hospitalisations in the remotely monitored groups[16]. The EFFECT study, a multi-centrer clinical trial, showed that remote monitoring of ICD in HF patients reduced mortality and cardiovascular hospitalisations [17], and the COMMIT-HF trial showed that remote monitoring of ICD/CRT HF patients significantly reduces long-term mortality but not HF-related hospitalisations [18].

**Table 2:** Remote monitoring in heart failure (*HF*) patients using implantable cardioverter defibrillator/cardiac resynchronisation therapy (*ICD/CRT*) devices

Trial/study	Author; journal; year	No. of patients	Parameter	Endpoint	Impact on HF hospitalisation
DOT-HF [16]	Van Veldhuisen et al.; Circulation; 2011	335	Intrathoracic impedance	HF hospitalisation	Increased
OptiLink [14]	Brachmann et al.; Eur J Heart Fail; 2011	1002	Intrathoracic impedance	HF hospitalisation	Non-significant
EFFECT [17]	De Simone et al.; Europace; 2015	987	Remote monitoring via ICD, or CRT	HF hospitalisation	Reduced (IRR 0.54; 95% CI 0.24–0.62)
MORE-CARE [13]	Boriani et al.; Eur J Heart Fail; 2017	865	Remote monitoring of advanced diagnostics via CRT‑D	HF hospitalisation	Non-significant
REM-HF [15]	Morgan et al.; Eur Heart J; 2017	1650	Remote monitoring via ICD, or CRT	HF hospitalisation	Non-significant
COMMIT-HF [18]	Kurek et al.; J Cardiovasc Electrophysiol; 2017	574	Remote monitoring via ICD, or CRT	HF hospitalisation/ All-cause mortality	Non-significant/ Reduced all-cause mortality (HR 0.24; 95% CI 0.14–0.41)
IN-TIME [19]	Hindricks et al; Lancet; 2014	716	Remote monitoring via ICD, or CRT	HF worsening score	OR 0.63 95% Ci 0.43–0.90

Other patient outcomes have been investigated as well, with mixed results. The IN-TIME RCT showed that using the remote monitoring abilities of the ICD and CRT devices leads to a reduction of a combined endpoint of all-cause death, overnight HF-related hospitalisation, change in New York Heart Association (NYHA) class, and change in patient global self-assessment [19]. However, other trials found no significant effect on patient outcomes [20, 21]. The effect of remote monitoring using ICD/CRT devices has recently been investigated in a meta-analysis, including 11 RCTs (5702 patients). This meta-analysis showed a reduction in the number of outpatient visits in remotely monitored patients, although remote monitoring with an ICD/CRT device had no effect on mortality or HF-related hospitalisations rates in these patients [22].

The MultiSENSE algorithm aims to predict the individualised risk for worsening of HF based on first and third heart sounds, thoracic impedance, respiration rate, the ratio of respiration rate to tidal volume, heart rate and patient activity. This could aid in the timely detection of HF worsening with the threshold retrospectively calculated by the algorithm. However, the overall sensitivity is only 70% [23]. Another algorithm with a similar aim is the HeartLogic algorithm [24]. To date, no clinical endpoint data or trial data are available and the technique is limited to certain ICD types and brands only.

### The shift in remote HF care: haemodynamic (invasive) remote monitoring

**Fig. 1:**
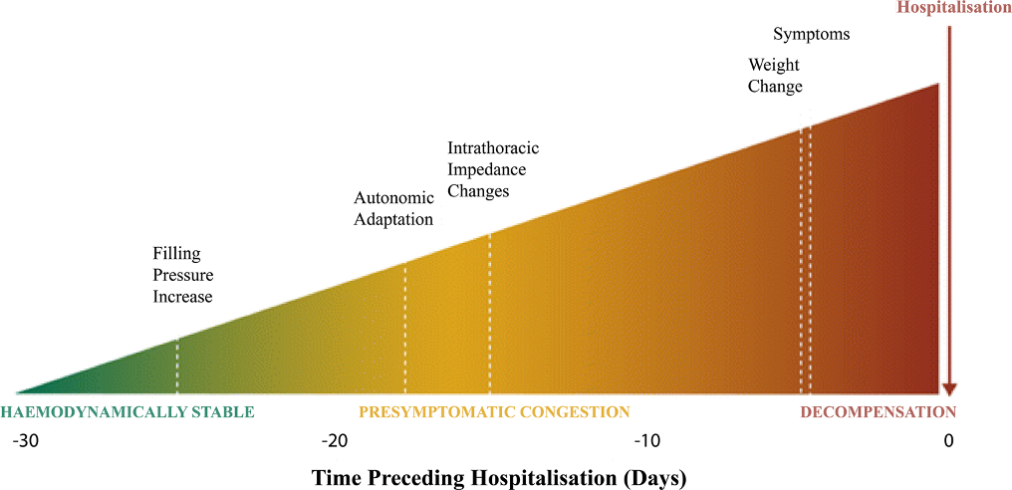
Pathophysiology of decompensated heart failure. (Reprinted from [54], with permission)

In HF patients cardiac filling pressures rise weeks before an exacerbation of HF leading to a related hospitalisation. Symptoms of clinical congestion such as gain in body weight will occur about 2 weeks later, usually shortly before hospitalisation (Fig. 1; [25]). Monitoring of cardiac filling pressures can be an effective strategy to detect upcoming HF decompensation, as it might provide a window of opportunity to intervene adequately and promptly, which is not possible with previous remote monitoring strategies. Therefore multiple implantable haemodynamic monitoring devices have been developed over the last few years. The ePAD (Medtronic, Dublin, Irland) device, an estimate pulmonary artery (PA) end-diastolic pressure device, can be implanted in the right ventricle and has been investigated in the COMPASS-HF trial. In this trial, NYHA class III/IV chronic HF patients were included and investigated as to whether remote haemodynamic monitoring using the ePAD could reduce HF-related hospitalisation, emergency or urgent care visits requiring intravenous therapy. This study did not find significant differences in its endpoint, although it was underpowered due to a lower inclusion rate. Furthermore, clinicians did not receive guidance on how to react to pressure changes [26].

Left atrium pressures (LAP) can be directly measured using a LAP device. The tip of this device is implanted transvenously into the atrial septum oriented towards the left atrium, enabling remote LAP monitoring. This device was used only in the LAPTOP-HF trial, which aimed to investigate the safety and effectiveness of this sensor. However, the enrollment was stopped early due to a perceived excess of procedure-related complications. This is an important issue as the procedure needs an interatrial septum puncture and is placed in the left side of the heart with the risk of arterial side complications. However, in the patients already included in this trial, and followed for 12 months, a 41% reduction of HF-related hospitalisations was observed in the patients with a LAP device [27]. Currently, the V‑LAP™ Left Atrium Monitoring systEm for Patients With Chronic sysTOlic and Diastolic Congestive heaRt Failure (VECTOR-HF) trial is investigating a new LAP device (V-LAP; Vectorious Medical Technologies Ltd., Tel Aviv, Israel) to assess the safety, performance and usability of this device in NYHA class III HF patients (NCT03775161).

**Fig. 2:**
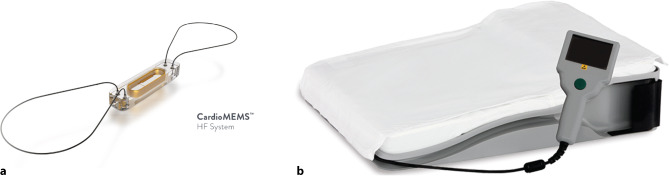
CardioMEMS HF system, consisting of the pulmonary artery pressure sensor (**a**) and the patient electronics system (**b**) used to take daily pressure readings. (Courtesy of Abbott, Inc.)

Off all the remote monitoring strategies currently available, remote haemodynamic monitoring using the CardioMEMS HF system device (Abbott Inc., Atlanta, GA, USA) (Fig. 2) appears to be the most promising with respect to safety, durability and ability to prevent HF-related hospitalisations. The CardioMEMS is implanted into the PA and enables daily pulmonary artery pressure (PAP) readings. Treating physicians can react to these changes in PA trend data to maintain normal PAP levels, as a sign of a stable clinical status. Furthermore, these daily PAP readings can be used as a feedback mechanism after treatment changes, providing feedback on whether the treatment changes led to a sufficient decline of PAPs. These strategies can lead to individualised HF therapy.

The CardioMEMS consists of a coil combined with a pressure-sensitive capacitor sealed in a capsule, forming an electrical circuit that resonates at a specific frequency when it is electromagnetically coupled with an external antenna [28]. This antenna provides the power for the device, so the device is completely free from batteries or leads. At both sides of the capsule, a loop is placed to ensure that the CardioMEMS remains at the implanted position until the endothelialisation is complete, approximately 3–4 weeks after implantation. When pressure is applied, the resonant frequency changes via a characteristic pattern and is received by the external antenna. The antenna converts this signal into a pressure waveform and sends it to a secure website, where it can be monitored. The device is implanted during a right heart catheterisation, with access via the femoral vein. An appropriate target location, based on vessel size and location, is identified on a pulmonary arteriogram. The CardioMEMS delivery system is advanced to the target location over a guidewire, where the CardioMEMS is released. After implantation, the device is calibrated using PAP obtained with a Swan Ganz catheter.

Two studies have validated the PAP measured by the CardioMEMS, with cardiac filling pressures measured by Swan-Ganz catheterisation or echocardiography directly after implantation and after 6 months of follow-up [29, 30]. Swan-Ganz measurements showed a good correlation with mean PAP assessed by CardioMEMS (*r*^2^ = 0.90 at implantation and *r*^2^ = 0.94 at follow-up, *p* < 0.01) [30]. Furthermore, a good correlation (*r*^2^ = 0.80 at implantation and *r*^2^ = 0.75, both *p* < 0.01 at follow-up) was found between PAP measurements by the CardioMEMS and estimated pressure measurements by echocardiography [29].

### Safety

The safety of the CardioMEMS has been investigated in the CHAMPION trial. A total of 15 serious adverse events occurred during all implantation attempts in the CHAMPION trial [31]. In total, 1% (*n* = 8) of patients developed a device-related adverse event, and 1% (*n* = 7) developed a procedure-related adverse event. The following events were reported: four bleeding events, three anticoagulation-related hospitalisations, two pre-existing atrial dysrhythmia exacerbations during implantation, two febrile illnesses, one pulmonary in situ thrombus during implantation that was treated with anticoagulation, one cardiogenic shock, one case of atypical chest pain, and one delivery-system failure requiring a snare to remove the delivery system [32]. An analysis of the post-marketing data of more than 5500 CardioMEMS implantations showed that 2.8% of all CardioMEMS patients experienced an adverse event [33]. Most adverse events were a recalibration of the system (*n* = 35) or access-site-related bleeding (*n* = 15). The reported adverse event rates are comparable with those of a standard right heart catheterisation, which is considered a safe procedure [34]. The recent US Post Approval Study (PAS) reported a device- or system-related complication in 0.3% of all patients, and a sensor failure in only 0.1% of all patients, which confirms the safety and durability of this technique.

### Clinical efficacy

The CardioMEMS was investigated for the first time in the CHAMPION trial [32]. In this trial, 550 patients with NYHA class III HF and at least one hospitalisation in the last year received a CardioMEMS and were randomised. Of the patients in the intervention group, the haemodynamic information was available to the treating physicians, and the physicians were instructed to react on pressure changes. In the control group, the CardioMEMS readings were not available to the physicians, and these patients received only the standard care. Using the haemodynamic feedback in the intervention group led to a significantly higher number of medication changes, especially diuretics and vasodilator changes, compared to the control group [35]. Furthermore, remote monitoring with the CardioMEMS device led to a significant reduction in mean PAP [32, 36]; similar results were observed in a real-world setting [37].

**Fig. 3:**
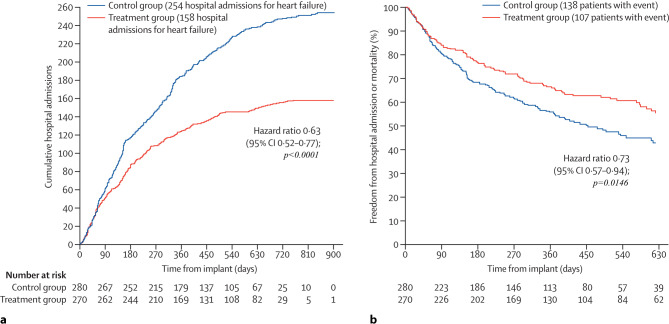
Cumulative heart-failure-related hospitalisations during the entire period of randomised single-blind follow-up (**a**), and freedom from first heart-failure-related hospitalisation or mortality (**b**) in the CHAMPION trial. (Reprinted from [32], with permission)

The effectiveness of the CardioMEMS in preventing HF-related hospitalisations has been investigated in multiple studies ([32, 36, 38, 39]; Fig. 3). During the first 6 months of remote monitoring of HF patients, the HF hospitalisation rates declined by approximately 30% [32, 38] compared with standard care. During the long-term follow-up, the sustained reduction was approximately 33% [31, 38, 39]. Also, all-cause hospitalisation rates were reduced: 45% at 6 months [38] and 16% at 18 months of follow-up [31]. None of these studies were powered to observe mortality differences; however, the CHAMPION trial showed a strong trend towards survival benefit in HF and reduced ejection fraction (HFrEF) patients monitored with the CardioMEMS system (*p* = 0.06) [40].

The recently presented PAS results confirm the consistent treatment benefit with CardioMEMS in chronic HF patients, reducing the number of HF hospitalisations in a more contemporary setting. The PAS study showed a 58% reduction in HF-related hospitalisation in the first year after CardioMEMS implantation compared with 1 year before implantation. Furthermore, a reduction in HF hospitalisations, mortality and all-cause mortality was observed after CardioMEMS implantation. However, patients included in the PAS study were their own historical controls and there has been no randomised comparison to standard care without PA monitoring.

### CardioMEMS and evidence in HFpEF patients

In a real-world setting, remote monitoring using the CardioMEMS leads to a similar reduction in mean PAP in both HFrEF and HFpEF patients [37]. Interestingly, in the CHAMPION trial, a larger reduction of HF-related hospitalisations in HFpEF patients compared with HFrEF patients was observed after at least 6 months of remote monitoring [40, 41]. Besides the alleged benefit of spironolactone in the United States (US) and European participants of the TOPCAT trial [42] with spironolactone, this is the first evidence of a treatment or tool to improve the outcome in HFpEF patients.

### Standard care in chronic HF

**Fig. 4:**
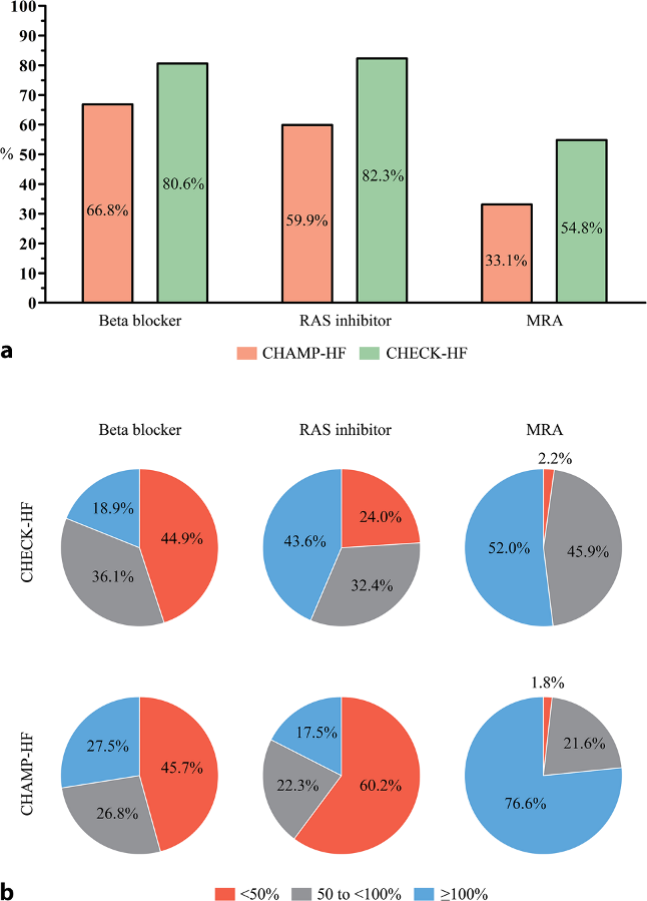
Differences between the United States and the Netherlands in the use of (**a**) and dosing of (**b**) guideline-recommended medication in patients with heart failure and reduced ejection fraction in the CHAMP-HF [43] and CHECK-HF [44] registries. *MRA* mineralocorticoid receptor antagonist (Adapted from [43, 44], with permission)

Recently two large HF registries have been published, the CHAMP-HF registry [43] from the USA and the CHECK-HF Registry [44] from The Netherlands. These two registries showed the differences in standard care between the USA and Western Europe. The prescription rates of RAS inhibitors (82.3% vs 59.9%), beta blockers (80.6% vs 66.8%) and mineralocorticoid receptor antagonist (MRA) (54.8% vs 33.1%) in HFrEF patients were much higher in the CHECK-HF registry compared to the CHAMP-HF registry (Fig. 4a). Furthermore, the prescribed dosages differed between the two registries, with higher prescribed dosages for RAS inhibitors in the CHECK-HF registry and higher dosages for MRA in the CHAMP-HF registry (Fig. 4b) Differences in the HF readmission rates were observed between the USA and Europe [45, 46]. The generalisability of the US findings in terms of clinical effectiveness when using the CardioMEMS device in addition to standard care is therefore limited, and additional costs cannot be directly extrapolated between the two different health care structures. Additional research is needed in the European setting. In Germany, the CardioMEMS European Monitoring Study for Heart Failure (MEMS-HF) study was set up as a post-marketing study to test the safety and clinical effectiveness in a European setting but lacks a control group [47].

### Cost-effectiveness of CardioMEMS

The cost-effectiveness of remote monitoring using the CardioMEMS is highly relevant. Using the US CHAMPION trial data the incremental cost-effective ratio (ICER, cost per quality-adjusted life-year) for the US setting has been calculated [32, 48–50]. These studies estimated an increase in the quality-adjusted life-years in the CardioMEMS group of between 0.28 and 0.58, with incremental costs between $4282 and $20,079, compared with standard care patients. This results in an estimated ICER in the USA of between $13,379 and $71,462, which are additional costs in order to gain one quality-adjusted life-year in patients monitored with the CardioMEMS device. Sensitivity analyses demonstrated that the cost-effectiveness of the CardioMEMS is highly influenced by device costs, costs of routine outpatient care, hospitalisation rates, mortality rates and duration of remote monitoring using the CardioMEMS.

There are no patient-level data for cost-effectiveness analyses in Western Europe. With assumptions and estimations based on extrapolating data from the CHAMPION trial and despite the large differences in standard care and financial systems, Cowie et al. [51] calculated the ICER in the European setting, which was approximately between €22,555 (for the Netherlands) and €23,814 (for Germany). However, all these analyses used data on the reduction of HF hospitalisation from the CHAMPION trial and used different estimated mortality rates from population-based cohorts for the cost-effectiveness analyses.

### Health care utilisation

Two studies investigated the potential reduction of health care utilisation achieved by using the CardioMEMS [38, 52]. In a real-world Medicare database, 1‑year remote monitoring with the CardioMEMS led to an $11,260 cost reduction for HF hospitalisations compared with 1 year before the CardioMEMS implantation [38]. Based on the effects reported in the CHAMPION trial, and the expected prevalence and hospitalisation costs in Germany, remote monitoring with the CardioMEMS could lead to an overall cost reduction of €106,000,000 in Germany in 2021 [52].

As shown above, remote monitoring of PAP with the CardioMEMS in chronic HF patients leads to more medication changes and a larger reduction of PAP compared with patients receiving standard care, indicating that these patients receive more individualised HF care. In the US, this strategy was effective in reducing the number of HF-related and all-cause hospitalisations. It was suggested that this strategy could improve mortality rates and has been shown to be cost-effective. However, as discussed earlier, some important differences in HF care exist between the USA and Europe.

### Recommendation of ESC 2016 guidelines on remote monitoring

The 2016 ESC guidelines report on the lack of consistent evidence for non-haemodynamic telemonitoring or remote monitoring in HF patients. The guidelines state that remote monitoring may be considered in selected patients to improve HF outcome with individual approaches such as CardioMEMS to reduce the risk of HF admissions and multi-parameter monitoring with ICD (in-time approach) to improve outcome in HFrEF patients with a level IIb class B recommendation [53].

## Conclusion

In recent years, many remote monitoring strategies have been developed, and development continues at a rapid rate. Non-invasive remote monitoring of symptoms and signs, as well as weight, has not been proven to be effective in improving outcome measurements. Also, the monitoring of biomarkers or thoracic impedance has not been shown to be beneficial. Invasive or haemodynamic measures of remote monitoring are developed with right-sided (CardioMEMS) and left-sided (LA devices) sensors. The LAPTOP-HF trial with LA devices was stopped early for safety reasons. The CardioMEMS is the most promising (invasive) remote monitoring tool currently available. The haemodynamic information allows for a window of timely and adequate intervention based upon raised PAP, preventing an upcoming HF decompensation. Additionally, its safety and durability have been tested and confirmed in post-marketing studies. However, important information on the effect on the quality of life and cost-effectiveness is still lacking in a Western European setting, which is currently being investigated in the MONITOR-HF study.
